# A pan-European survey of robotic training for gastrointestinal surgery: European Robotic Surgery Consensus (ERSC) initiative

**DOI:** 10.1007/s00464-024-11373-x

**Published:** 2024-12-04

**Authors:** Michael G. Fadel, Josephine Walshaw, Francesca Pecchini, Marina Yiasemidou, Matthew Boal, Muhammed Elhadi, Matyas Fehervari, Lisa H. Massey, Francesco Maria Carrano, Stavros A. Antoniou, Felix Nickel, Silvana Perretta, Hans F. Fuchs, George B. Hanna, Christos Kontovounisios, Nader K. Francis, the European Robotic Surgery Consensus (ERSC) study group

**Affiliations:** 1 Department of Surgery and Cancer, Imperial College, London, UK; 2 Leeds Institute of Medical Research, St James’s University Hospital, University of Leeds, Leeds, UK; 3 Division of General Surgery, Emergency and New Technologies, Baggiovara General Hospital, Modena, Italy; 4 The Royal London Hospital, Barts Health NHS Trust, London, UK; 5 The Griffin Institute, Northwick Park and St Mark’s Hospital, London, UK; 6 Tripoli University Hospital, Tripoli, Libya; 7 Bariatric Surgery Department, Maidstone and Tunbridge Wells NHS Trust, Kent, UK; 8 Department of Colorectal Surgery, Nottingham University Hospitals NHS Trust, Nottingham, UK; 9 Department of Medical and Surgical Sciences and Translational Medicine, Faculty of Medicine and Psychology, St Andrea Hospital, Sapienza University, Rome, Italy; 10 Department of Surgery, Papageorgiou General Hospital, Thessaloniki, Greece; 11 Department of General, Visceral and Thoracic Surgery, University Medical Center Hamburg-Eppendorf, Hamburg, Germany; 12 IRCAD, Research Institute Against Digestive Cancer, Strasbourg, France; 13 NHC University Hospital, Strasbourg, France; 14 Department of General, Visceral, Cancer and Transplantation Surgery, University Hospital Cologne, Cologne, Germany; 15 Department of Colorectal Surgery, Chelsea and Westminster Hospital NHS Foundation Trust, London, UK; 16 Department of Colorectal Surgery, Royal Marsden NHS Foundation Trust, London, UK; 17 2nd Surgical Department, Evaggelismos Athens General Hospital, Athens, Greece

**Keywords:** Robotic surgery, Gastrointestinal surgery, Training, Certification, Assessment

## Abstract

**Background:**

There has been a recent rapid growth in the adoption of robotic systems across Europe. This study aimed to capture the current state of robotic training in gastrointestinal (GI) surgery and to identify potential challenges and barriers to training within Europe.

**Methods:**

A pan-European survey was designed to account for the opinion of the following GI surgery groups: (i) experts/independent practitioners; (ii) trainees with robotic access; (iii) trainees without robotic access; (iv) robotic industry representatives. The survey explored various aspects, including stakeholder opinions on bedside assisting, console operations, challenges faced and performance assessment. It was distributed through multiple European surgical societies and industry, in addition to social media and snowball sampling, between December 2023 and March 2024.

**Results:**

A total of 1360 participants responded, with valid/complete responses from 1045 participants across 38 European countries. Six hundred and ninety-five (68.0%) experts and trainees were not aware of a dedicated robotic training curriculum for trainees, with 13/23 (56.5%) industry representatives not incorporating training for trainees in their programme. Among trainees with access to robotic systems, 94/195 (48.2%) had not performed any robotic cases, citing challenges including a lack of certified robotic trainers and training lists. Both experts and trainees agreed that trainees should start bedside assisting and operating on the console earlier than they currently do. Assessment tools of trainee performance were not being used by 139/479 (29.0%) participants.

**Conclusion:**

This pan-European survey highlights the need for a standardised robotic curriculum to address the gap in visceral training, assessment and certification. A greater emphasis may be required on implementing robotic training earlier through simulation training, dual console learning, bedside assisting, key clinical performance indicators, and assessment tools. The findings will guide the development of a pan-European consensus on the essential components of a comprehensive training programme for GI robotic surgery.

**Supplementary Information:**

The online version contains supplementary material available at 10.1007/s00464-024-11373-x.

The adoption of robotic systems in general surgery has rapidly increased over the past three decades [1, 2]. The Da Vinci robotic system (Intuitive Surgical Inc., California, USA) was the first to gain Food and Drug Administration (FDA) approval as early as 2000 [3]. Since then, there have been advancements in robotic technologies, with now over 20 existing robotic platforms globally [4]. These systems offer a three-dimensional visual field and articulated instruments that provide seven degrees of motion, enhancing the surgeon’s ability to perform operations with greater precision [5, 6]. Reduced physiological tremor, fatigue, and musculoskeletal strain may also help improve concentration and performance [7].

With the initiation of this new technology, surgeons must have sufficient training to ensure that they perform these techniques competently and safely. Gaining robotic aptitude involves a unique learning curve that is significantly different from traditional laparoscopy [8]. This includes elements accounting for endowristed instrument handlings, three-dimensional visualisation, four-arm usage including the camera, and lack of haptic feedback [9–11]. Furthermore, the inclusion of a robot to the team adds further complexity to the operation and increases demands on the entire theatre team [12]. To reduce the learning curve, trainees can initially be exposed to robotic training via several methods, including simulated surgical training (dry lab), cadaveric and animal models (wet labs), and virtual reality simulation [13]. This should be followed by documented experience and supervision of an appropriate volume of cases with satisfactory outcomes [14–18].

Currently, the integration of robotics is not uniform across surgical specialties and training programmes internationally, which can result in significant variability in training opportunities. The education and learning opportunities offered to robotic surgeons vary in content and design, and the quality of training differs depending on region and country [19–22]. Some robotic companies provide in-house training programmes for experienced general surgeons (e.g. Da Vinci Technology Training Pathway), however, many of these programmes do not specifically address the needs of trainees. Therefore, the steps of progression and the minimum level of robotic training that is required for trainees to achieve accreditation remain unclear.

The European Robotic Surgery Consensus (ERSC) study group, supported by the European Association for Endoscopic Surgery (EAES), has been established to reach a European consensus on a robotic curriculum for gastrointestinal (GI) trainees, which aims to improve training, and ultimately enhance patient safety and outcomes [23]. In line with the registered protocol [24], the study group have created a comprehensive pan-European survey to capture the current state of robotic training and identify potential challenges and solutions for GI trainees. We will also compare the responses from GI experts and trainees with robotic access to identify and assess any potential differences in their views on robotic training. The results of this survey will, in turn, inform the development of a robotic training curriculum for GI surgery trainees.

## Methods

The ERSC Steering Committee developed a pan-European survey following regular meetings until informal consensus was achieved for a final set of questions to be included. The domains of interest that were discussed included robotic simulation, courses, bedside assisting, performed cases, mentorship, and assessment. The survey was hosted on Qualtrics XM software [25], a secure web-based survey platform for response collection. The questionnaire was tested and validated through dissemination to a small group of experts and non-experts, including the usability and technical functionality, before fielding the survey. The period of online collection was approximately three months (19th December 2023 to 24th March 2024). The study was reviewed by our Institutional Review Board and deemed not to require ethical approval. Data collection and analysis were anonymised according to General Data Protection Regulation (GDPR) legislation. Participation was voluntary and all individuals could withdraw at any time before the completion and submission of the survey.

The survey initially consisted of demographic questions followed by a number of questions for each of the four target groups below in a branching format:

(i) Experts (defined as an independent practitioner) with and without robotic access (19 questions)
(ii) Trainees with robotic access (25 questions)
(iii) Trainees without robotic access (10 questions)
(iv) Robotic industry representatives (7 questions).

The survey explored various aspects, including demographic information, stakeholder opinions on the optimal timing for GI trainees to begin bedside assisting and console operations, methods for trainee assessment, and the minimum requirements for achieving competency for a procedure (defined as reaching or surpassing the learning curve). It also asked trainees about current access to robotic training, challenges faced, and how surgical societies can support and enhance their training. Participants had the option of selecting multiple responses for some questions, and they had the ability to review using a back button and change their answers if necessary prior to submission. The survey questions and the total number of responses received for each question are shown in Supplementary Material S1.

The survey was specifically designed for European countries, and we have only included the responses from European visceral experts and trainees in the analysis. Responses from global industry providers were also included to gain insights into their current policies and assessment pathways. Partial responses were excluded if participants only provided responses in the demographics section, without providing any results in their relevant branching section. These responses were deemed incomplete or invalid.

The survey was distributed via members of several European based societies such as EAES, United European Gastroenterology (UEG), Upper GI International Robotic Association (UGIRA), Association of Laparoscopic Surgeons of Great Britain and Ireland (ALSGBI), Società Italiana di Chirurgia Endoscopica (SICE), German Society of Surgery Section of Computer and Telematic Assisted Surgery (CTAC), Professional Association of German Surgery (BDC), Research Institute against Digestive Cancer (IRCAD), European Society of Coloproctology (ESCP), Belgian Robotic Surgery Working Group (RSWG), and British Obesity and Metabolic Surgery Society (BOMSS). Two separate survey invitations were sent via email approximately four weeks apart, where possible, to maximise the response rate. An example of the survey announcement disseminated through the EAES newsletter is provided in Supplementary Material S2. The survey was also distributed directly through robotic industry contacts and our X (formerly known as Twitter) page (@ERCS_Study).

The results of the survey have been reported in line with the Checklist for Reporting Results of Internet E-Surveys (CHERRIES) where applicable [26]. The CHERRIES checklist can be found in Supplementary Material S3. Response data were compiled and analysed using Microsoft Excel and IBM SPSS version 29.0.2. Categorical data are presented as frequencies and percentages, and continuous variables are presented as medians and ranges. Two group comparisons of data from experts and trainees with robotic access were made using the chi-squared test, where applicable, and statistical significance was defined as a *p* value < 0.05.

## Results

During the data collection time period, 1360 responses were received. After excluding non-GI and non-European responses (*n* = 101), and incomplete/invalid responses (*n* = 214), 1045 responses were analysed: 284 (27.2%) from experts with and without robotic access; 258 (24.7%) from trainees with robotic access, 480 (45.9%) from trainees without robotic access, and 23 (2.2%) from industry providers (Table 1). Respondents were from 38 European countries, with the top 5 contributing countries being Germany (*n* = 388; 37.1%), Italy (*n* = 172; 16.5%), United Kingdom (UK) (*n* = 144; 13.8%), Greece (*n* = 66; 6.3%), and France (*n* = 52; 5.0%). Eight hundred and eighty-seven (84.8%) participants were aged between 31 and 60 years old, and 289 (27.7%) were female participants. The majority of participants chose general surgery (*n* = 437; 41.8%) as their current specialty, along with colorectal (*n* = 230; 22.0%), upper GI (*n* = 115; 11.0%), and surgical oncology (*n* = 82; 7.8%). Seventy percent of responders were practising in a public teaching hospital, with 672 (64.3%) participants stating they had a robotic console in their current hospital. Where reported, approximately half of the trainees were seven years into their postgraduate surgical training.

**Table 1:** Demographics of survey participants

Demographics	Category	Participants, *n* (%)
Age, years	21–30	83 (7.9)
	31–40	397 (38.0)
	41–50	256 (24.5)
	51–60	234 (22.4)
	61–70	73 (7.0)
	71–80	2 (0.2)
Gender	Male	749 (71.7)
	Female	289 (27.7)
	Other	7 (0.7)
Ethnicity	White	823 (78.8)
	Asian	72 (6.9)
	Arab	38 (3.6)
	Black	16 (1.5)
	Mixed	15 (1.4)
	Other	81 (7.8)
Surgical sub-specialty	General surgery	437 (41.8)
	Colorectal	230 (22.0)
	Upper GI	115 (11.0)
	Surgical oncology	82 (7.8)
	Hepatobiliary	61 (5.8)
	Bariatric	60 (5.7)
	Other	60 (5.7)
Top contributing countries	Germany	388 (37.1)
	Italy	172 (16.5)
	UK	144 (13.8)
	Greece	66 (6.3)
	France	52 (5.0)
	Romania	44 (4.2)
	Spain	16 (1.5)
	Switzerland	15 (1.4)
	Turkey	12 (1.1)
	Austria	10 (1.0)
	Belgium	9 (0.9)
	Portugal	9 (0.9)
	Malta	7 (0.7)
	Bulgaria	6 (0.6)
Hospital type	Public (teaching)	739 (70.7)
	Public (non-teaching)	107 (10.2)
	Private (teaching)	120 (11.5)
	Private (non-teaching)	52 (5.0)
	Other	27 (2.6)
Robotic console in current hospital	Yes	672 (64.3)
	No	360 (34.4)
	Not sure	13 (1.2)
GI surgery group	Expert/independent surgeon	284 (27.2)
	Trainee with robotic access	258 (24.7)
	Trainee without robotic access	480 (45.9)
	Industry provider	23 (2.2)
Postgraduate year of surgical training	1–2	46 (8.7)
	3–4	99 (18.7)
	5–6	111 (20.9)
	7	250 (47.2)
	Other	24 (4.5)

In total, 695 (68.0%) participants stated they were not aware of a dedicated training curriculum to achieve accreditation in robotic surgery in their country. Of those, 592 (85.2%) believed that a dedicated training curriculum for robotic surgery should become mandatory if the infrastructure is in place.

### Views on the robotic training pathway from experts and trainees with robotic access

**Fig. 1:**
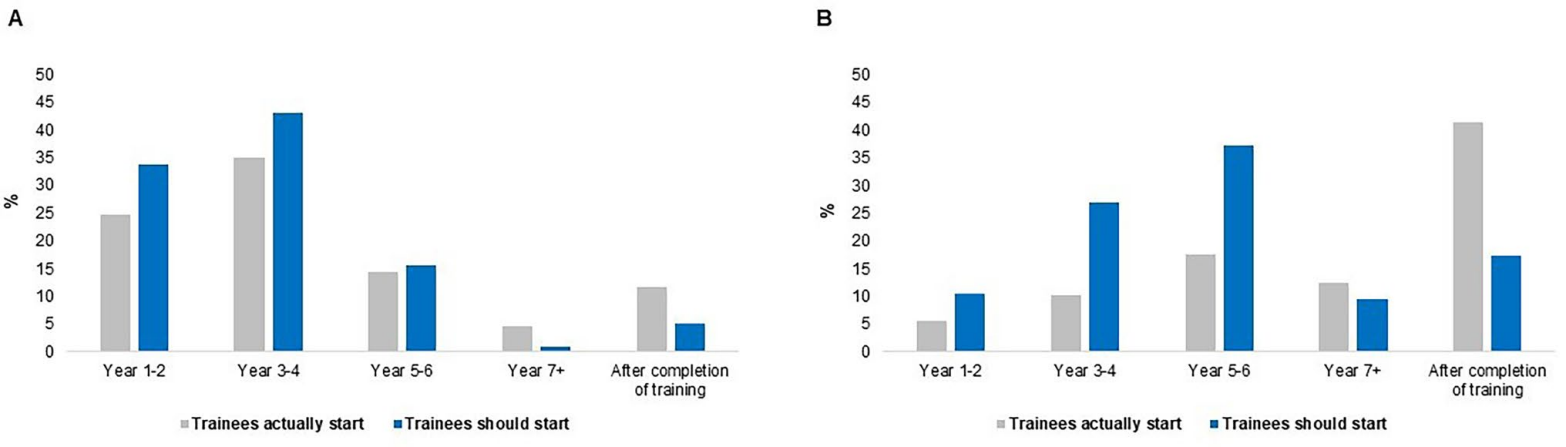
Responses from experts and trainees with robotic access on **A** what level do trainees actually start versus should start bedside assisting and **B** what level do trainees actually start versus should start operating on the console

The majority of participants had the Da Vinci robotic system in their institution (*n* = 458; 85.9%), followed by Hugo (*n* = 32; 6.0%) and Versius (*n* = 28; 5.3%) systems (Table 2). Four hundred and forty-four (81.9%) ‘strongly agreed’ or ‘agreed’ with the statement that a dual robotic console is essential for training. In addition, a large proportion of trainees (*n* = 143; 73.0%) found online e-learning robotic training modules useful. Both experts and trainees agreed that trainees should start virtual reality simulation as early as possible, ideally within the first 4 years of surgical training (*n* = 339; 70.8%; *p* = 0.929). Two hundred and sixty-four (93.0%) experts felt bedside assisting training was essential for trainees prior to operating on the console. A considerable number of experts (*n* = 92; 34.8%) also believed that bedside assisting for at least 11–20 cases was sufficient to start operating on the robotic console. Experts and trainees agreed that trainees should start bedside assisting (*p* = 0.764) (Fig. 1A) and operating on the console earlier than they currently do (*p* = 0.350) (Fig. 1B).

**Table 2:** Survey responses from experts/independent surgeons, trainees with and without robotic access, and industry providers on robotic curricula components

	Expert/independent surgeon, *n* = 284 *n* (%)	Trainees with robotic access, *n* = 258 *n* (%)	Trainees without robotic access, *n* = 480 *n* (%)	Industry provider, *n* = 23 *n* (%)	Total *n* (%)	*p*-value (experts vs robotic trainees with access)
Robotic console and training pathway						
Which robot do you have in your institution?						
Da Vinci	219 (79.6)	239 (92.6)	**–**	**–**	458 (85.9)	–
Hugo	16 (5.8)	16 (6.2)			32 (6.0)	
Versius	15 (5.5)	13 (5.0)			28 (5.3)	
Dexter	5 (1.8)	4 (1.6)			9 (1.7)	
Senhance	4 (1.5)	2 (0.8)			6 (1.1)	
Other	16 (5.8)	12 (4.7)			28 (5.3)	
Do you think a dual robotic console is essential for training?						
1—Strongly disagree	12 (4.2)	4 (1.6)	**–**	**–**	16 (3.0)	0.061
2—Disagree	15 (5.3)	7 (2.7)			22 (4.1)	
3—Neither agree nor disagree	37 (13.0)	23 (8.9)			60 (11.1)	
4—Agree	80 (28.2)	80 (31.0)			160 (29.5)	
5—Strongly agree	140 (49.3)	144 (55.8)			284 (52.4)	
If you have a training pathway, which of the following do you use?						
Da Vinci Technology Training Pathway	219 (79.3)	187 (72.5)	**–**	**–**	406 (76.0)	–
FRS	18 (6.5)	38 (14.7)			56 (10.5)	
FSRS	22 (8.0)	27 (10.5)			49 (9.2)	
Other	17 (6.2)	8 (3.1)			25 (4.7)	
Online e-learning and virtual reality						
Do you find online e-learning robotic training modules useful?						
1—Strongly disagree	–	5 (2.6)	**–**	**–**	5 (2.6)	–
2—Disagree		11 (5.6)			11 (5.6)	
3—Neither agree nor disagree		37 (18.9)			37 (18.9)	
4—Agree		70 (35.7)			70 (35.7)	
5—Strongly agree		73 (37.2)			73 (37.2)	
When do you think trainees should start virtual reality training?						
Year 1–2	90 (31.7)	67 (34.4)	**–**	**–**	157 (32.8)	0.929
Year 3–4	109 (38.4)	73 (37.4)			182 (38.0)	
Year 5–6	48 (16.9)	29 (14.9)			77 (16.1)	
Year 7 +	4 (1.4)	3 (1.5)			7 (1.5)	
After completion of surgical training	22 (7.7)	18 (9.2)			40 (8.4)	
Not essential	11 (3.9)	4 (2.1)			15 (3.1)	
Other	0 (0.0)	1 (0.5)			1 (0.2)	
Robotic bedside assisting						
How many robotic cases have you bedside assisted in?						
0	–	13 (6.7)	**–**	**–**	13 (6.7)	–
1–10		50 (25.6)			50 (25.6)	
11–20		37 (19.0)			37 (19.0)	
21–30		31 (15.9)			31 (15.9)	
31–50		25 (12.8)			25 (12.8)	
> 50		39 (20.0)			39 (20.0)	
How many bedside assisting cases should surgeons have before operating on the console?						
1–10	37 (14.0)	–	**–**	**–**	37 (14.0)	–
11–20	92 (34.8)				92 (34.8)	
21–30	63 (23.9)				63 (23.9)	
31–50	56 (21.2)				56 (21.2)	
51–100	11 (4.2)				11 (4.2)	
> 100	5 (1.9)				5 (1.9)	
At what level do trainees start robotic bedside assisting in your institution?						
Year 1–2	79 (27.8)	39 (20.0)	**–**	**–**	118 (24.6)	–
Year 3–4	97 (34.2)	71 (36.4)			168 (35.1)	
Year 5–6	40 (14.1)	29 (14.9)			69 (14.4)	
Year 7 +	9 (3.2)	13 (6.7)			22 (4.6)	
After completion of surgical training	26 (9.2)	30 (15.4)			56 (11.7)	
Not applicable	28 (9.9)	0 (0.0)			28 (5.8)	
Other	5 (1.8)	13 (6.7)			18 (3.8)	
At what level should trainees start robotic bedside assisting at?						
Year 1–2	91 (32.0)	71 (36.4)	**–**	6 (31.6)	168 (33.7)	0.764
Year 3–4	127 (44.7)	83 (42.6)		5 (26.3)	215 (43.2)	
Year 5–6	45 (15.8)	27 (13.8)		6 (31.6)	78 (15.7)	
Year 7 +	2 (0.7)	3 (1.5)		0 (0.0)	5 (1.0)	
After completion of surgical training	15 (5.3)	10 (5.1)		1 (5.3)	26 (5.2)	
Other	4 (1.4)	1 (0.5)		1 (5.3)	6 (1.2)	
Operating on the robotic console						
How many robotic cases have you performed?						
0	18 (6.3)	94 (48.2)	–	–	112 (23.4)	< 0.001*
1–10	10 (3.5)	59 (30.3)			69 (14.4)	
11–20	23 (8.1)	16 (8.2)			39 (8.1)	
21–30	17 (6.0)	11 (5.6)			28 (5.8)	
31–50	37 (13.0)	9 (4.6)			46 (9.6)	
51–100	62 (21.8)	6 (3.1)			68 (14.2)	
> 100	117 (41.2)	0 (0.0)			117 (24.4)	
At what level do trainees start operating on the robotic console in your institution?						
Year 1–2	18 (6.3)	9 (4.6)	**–**	**–**	27 (5.6)	–
Year 3–4	29 (10.2)	20 (10.2)			49 (10.2)	
Year 5–6	60 (21.1)	25 (12.8)			85 (17.7)	
Year 7 +	38 (13.4)	22 (11.2)			60 (12.5)	
After completion of surgical training	96 (33.8)	103 (52.6)			199 (41.5)	
Not applicable	38 (13.4)	17 (8.7)			55 (11.5)	
Other	5 (1.8)	0 (0.0)			5 (1.0)	
At what level should trainees start operating on the robotic console in your institution?						
Year 1–2	26 (9.2)	24 (12.3)	**–**	1 (5.3)	51 (10.2)	0.350
Year 3–4	66 (23.2)	55 (28.2)		8 (42.1)	129 (25.9)	
Year 5–6	110 (38.7)	65 (33.3)		3 (15.8)	178 (35.7)	
Year 7 +	31 (10.9)	15 (7.7)		0 (0.0)	46 (9.2)	
After completion of surgical training	42 (14.8)	35 (17.9)		6 (31.6)	83 (16.7)	
Not applicable	5 (1.8)	0 (0.0)		0 (0.0)	5 (1.0)	
Other	4 (1.4)	1 (0.5)		1 (5.3)	6 (1.2)	

*FRS* Fundamentals of Robotic Surgery; *FSRS* Fundamental Skills of Robotic-Assisted Surgery. *statistically significant

### Views from trainees with and without robotic access

**Fig. 2:**
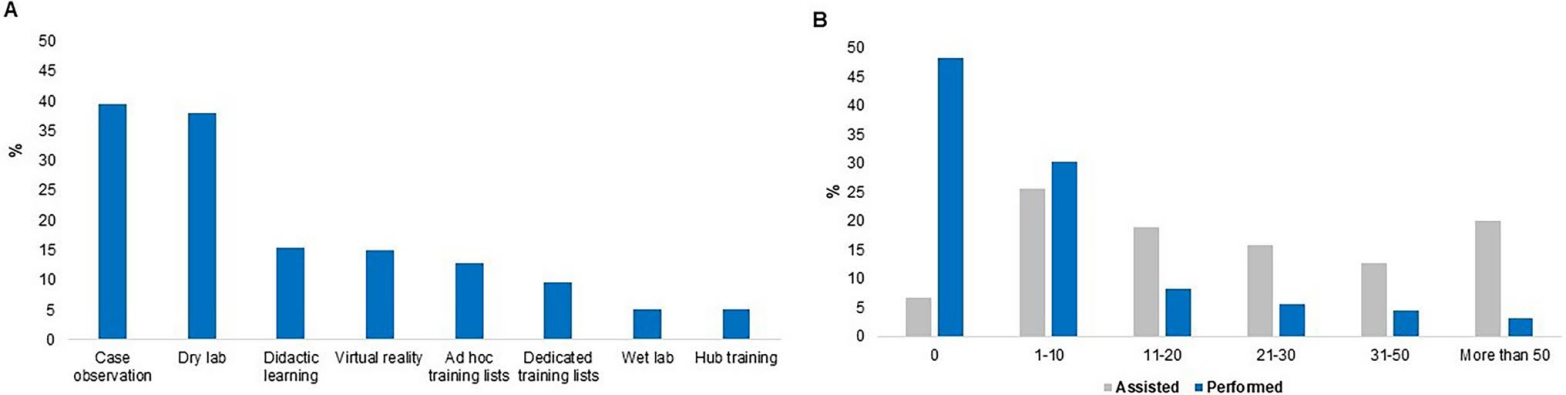
Responses from trainees with robotic access on **A** current access to robotic training methods and tools and **B** how many robotic cases have they bedside assisted in and performed

The most common robotic training methods available for trainees with robotic access were case observation (*n* = 77; 39.5%), dry lab (*n* = 74; 37.9%), and didactic learning (*n* = 30; 15.4%) (Fig. 2A). The least commonly available training methods were wet lab training (*n* = 10; 5.1%), in the form of cadaveric or animal models, and an accessible training hub or centre (*n* = 10; 5.1%). Regarding access to an on-site robotic simulator, only 83 trainees (32.2%) had access during normal working hours, and 67 trainees (10.1%) had no access to a robotic simulator at all. Twenty trainees (41.7%) with no access had to travel over an hour to reach their nearest robotic simulator. One hundred and thirty-eight (70.4%) trainees with access have attended a robotic training course, however, only 64 (32.8%) trainees had access to a dedicated robotic training fellowship at their institution.

A significant proportion of trainees without access stated they would like to learn how to use the robot (*n* = 454; 94.6%), preferably within the first 4 years of surgical training (*n* = 264; 55.0%). One hundred and forty trainees (29.2%) would be willing to travel more than 2 hours to reach their nearest robotic simulator. Overall, the main common challenges faced by trainees were a lack of certified robotic trainers (*n* = 356; 52.7%) and training lists (*n* = 348; 51.6%), and the cost of simulators and courses (*n* = 319; 47.3%) (Table 3). This was evidenced by the fact that 94 (48.2%) of trainees with access had not performed any robotic cases (Fig. 2B).

**Table 3:** Survey responses from experts/independent surgeons, trainees with and without robotic access, and industry providers on robotic training opportunities and challenges, mentorship and assessment of competency

	Experts/independent surgeon, *n* = 284 *n* (%)	Trainees with robotic access, *n* = 258 *n* (%)	Trainees without robotic access, *n* = 480 *n* (%)	Industry provider, *n* = 23 *n* (%)	Total *n* (%)	*p*-value (experts vs robotic trainees with access)
Robotic training access and challenges faced						
Do you have access to robotic training in your institution?						
Case observation	–	77 (39.5)	**–**	**–**	77 (39.5)	–
Dry lab		74 (37.9)			74 (37.9)	
Didactic learning		30 (15.4)			30 (15.4)	
Ad hoc training		29 (14.9)			29 (14.9)	
Virtual reality		25 (12.8)			25 (12.8)	
Dedicated training lists		19 (9.7)			19 (9.7)	
Wet lab		10 (5.1)			10 (5.1)	
Hub training		10 (5.1)			10 (5.1)	
None of the above		40 (20.5)			40 (20.5)	
How frequently do you receive robotic training in your hospital?						
More than one theatre session per week	–	22 (11.3)	**–**	**–**	22 (11.3)	–
One theatre session per week		30 (15.4)			30 (15.4)	
One theatre session per fortnight		19 (9.7)			19 (9.7)	
One theatre session per month or less		58 (29.7)			58 (29.7)	
Never		66 (33.8)			66 (33.8)	
Have you faced any challenges in gaining robotic training?						
Lack of robotic accredited trainers in hospital	–	74 (37.9)	282 (58.8)	–	356 (52.7)	–
Lack of robotic training lists		74 (37.9)	274 (57.1)		348 (51.6)	
Cost of simulator or courses		73 (37.4)	246 (51.3)		319 (47.3)	
Competition of training opportunities		101 (51.8)	184 (38.3)		285 (42.2)	
Insufficient hands-on exposure during robotic training		78 (40.0)	174 (36.3)		252 (37.3)	
Failure to progress from assisting to operating		68 (34.9)	126 (26.3)		194 (28.7)	
Great distance to travel to courses or institutions		40 (20.5)	117 (24.4)		157 (23.3)	
None		20 (10.3)	18 (3.8)		38 (5.6)	
Other		10 (5.1)	40 (8.3)		50 (7.4)	
Robotic training competency and mentorship						
How many cases do you think a trainee needs to perform to reach robotic surgery competency?						
1–10	2 (0.7)	–	–	0 (0.0)	2 (0.7)	–
11–20	36 (12.7)			2 (10.5)	38 (12.5)	
21–30	61 (21.5)			5 (26.3)	66 (21.8)	
31–50	106 (37.3)			6 (31.6)	112 (37.0)	
51–80	41 (14.4)			2 (10.5)	43 (14.2)	
81–100	23 (8.1)			1 (5.3)	24 (7.9)	
> 100	15 (5.3)			3 (15.8)	18 (5.9)	
Do you use tools to track or assess the performance of trainees in robotic surgery?						
Subjective assessment	142 (50.0)	56 (28.7)	–	–	198 (41.3)	< 0.001*
Case logs	96 (33.8)	77 (39.5)			173 (36.1)	
Video assessment	95 (33.5)	73 (37.4)			168 (35.1)	
Summative tools	29 (10.2)	30 (15.4)			59 (12.3)	
No assessment	74 (26.1)	65 (33.3)			139 (29.0)	
Other	8 (2.8)	3 (1.5)			11 (2.3)	
How should robotic surgery competency be assessed?						
Subjective assessment	196 (69.0)	107 (54.9)	288 (60.1)	14 (73.7)	605 (61.9)	0.004*
Case logs	136 (47.9)	102 (52.3)	252 (52.6)	15 (78.9)	505 (51.7)	
Video assessment	124 (43.7)	112 (57.4)	224 (46.8)	11 (57.9)	502 (51.4)	
Summative tools	124 (43.7)	122 (62.6)	319 (66.6)	14 (73.7)	579 (59.3)	
No assessment	6 (2.1)	9 (4.6)	11 (2.3)	0 (0.0)	26 (2.7)	
Other	4 (1.4)	8 (4.1)	0 (0.0)	3 (15.8)	15 (1.5)	
Who oversees your robotic training?						
The institution	135 (47.5)	64 (32.8)	**–**	**–**	199 (41.5)	–
Industry	69 (24.3)	37 (19.0)			106 (22.1)	
Regional training bodies	13 (4.6)	10 (5.1)			23 (4.8)	
Surgical societies	10 (3.5)	10 (5.1)			20 (4.2)	
National training bodies	8 (2.8)	6 (3.1)			14 (2.9)	
Not sure	39 (13.7)	54 (27.7)			93 (19.4)	
Other	10 (3.5)	14 (7.2)			24 (5.0)	
Who do you think should be overseeing robotic training for trainees?						
The institution	106 (37.3)	82 (42.1)	116 (24.2)	–	304 (31.7)	< 0.001*
Industry	4 (1.4)	3 (1.5)	9 (1.9)		16 (1.7)	
Regional training bodies	30 (10.6)	24 (12.3)	93 (19.4)		147 (15.3)	
Surgical societies	71 (25.0)	43 (22.1)	150 (31.3)		264 (27.5)	
National training bodies	63 (22.2)	40 (20.5)	103 (21.5)		206 (21.5)	
Other	10 (3.5)	3 (1.5)	9 (1.9)		22 (2.3)	

* = statistically significant

### Views on robotic training competency and mentorship from experts, trainees with and without robotic access

**Fig. 3:**
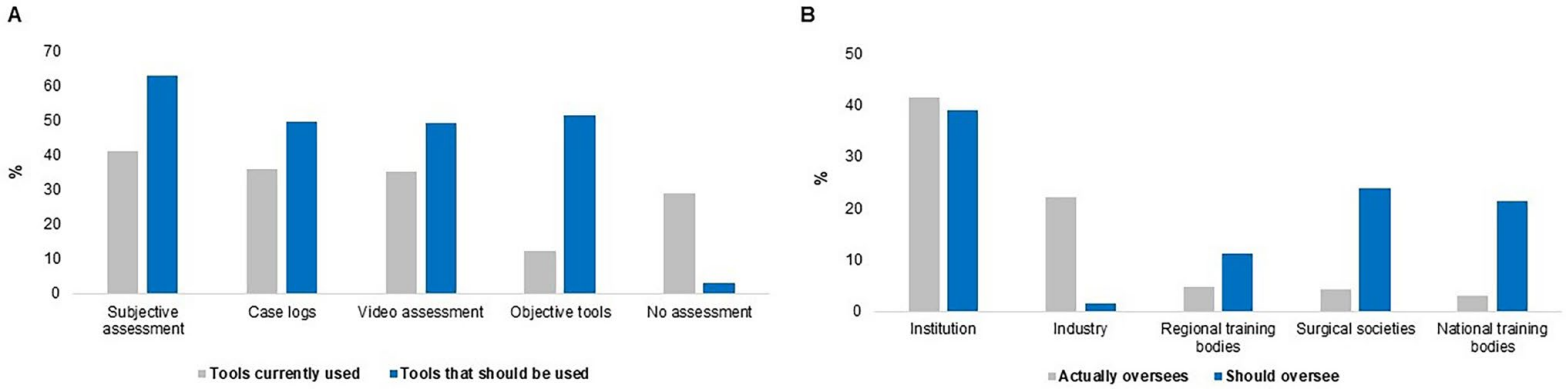
Responses from experts and trainees with robotic access on **A** which tools are currently being used versus which tools should be used to assess performance on the robotic console and **B** who actually oversees versus who should oversee robotic training

A considerable proportion of experts (*n* = 106; 37.3%) believed that trainees performing 31–50 cases was sufficient to reach the learning curve for a given procedure. A total of 139 (29.0%) participants were not currently using any tools to track or assess trainee performance. It was felt that summative assessment/objective tools should be adopted (*n* = 565; 59.0%) at a higher rate than they are currently being used (*n* = 59; 12.3%) (Fig. 3A). There were differences found in assessment tools usage between experts and trainees, with a greater proportion of experts using subjective assessment (50.0% versus 28.7%; *p* < 0.001). Experts believed that there should be a higher uptake in the use of multiple assessment tools compared to trainees with robotic access (*p* = 0.004). Only a minority of experts (2.1%) and trainees (4.6%) felt that an assessment tool is not required for robotic surgery training.

**Fig. 4:**
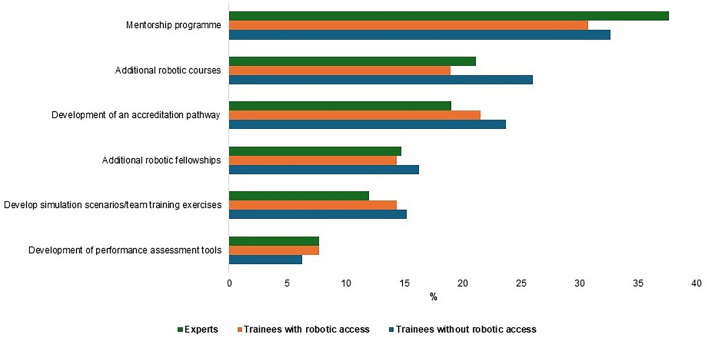
Experts, trainees with and without robotic access ranked as being the most important in response to how should surgical societies support robotic training

The survey results showed that participants preferred regional/national training bodies (*n* = 353; 36.8%) and surgical societies (*n* = 264; 27.5%) to oversee training rather than industry (*n* = 16; 1.7%) (Fig. 3B). Trainees with robotic access believed that the institution should have a greater oversight role in robotic training than trainees without robotic access did (42.1% versus 24.2%; *p* < 0.001). Approximately 20% of experts/trainees were not sure who currently provided oversight for their robotic training. In addition, we asked the first three target groups to rank how surgical societies should support the training of GI trainees, and the most desired suggestions were: (i) mentorship programmes, (ii) additional robotic courses, (iii) development of an accreditation pathway, (iv) additional robotic fellowships, and (v) development of simulation scenarios and team training exercises (Fig. 4 and Supplementary Material S4).

### Views from robotic industry representatives

We received responses from 23 industry representatives including Intuitive (*n* = 11; 47.8%), CMR Surgical (*n* = 2; 8.7%), Medtronic (*n* = 2; 8.7%), Distalmotion (*n* = 4; 17.4%), and Asensus (*n* = 2; 8.7%) representatives, along with Virtual Incision (*n* = 1; 4.3%), Stealth Autoguide (*n* = 1; 4.3%), and MiroSurge (*n* = 1; 4.3%). Thirteen (56.5%) industry representatives stated that they do not currently incorporate robotic training for GI trainees in their company training programme. From those that incorporate robotic training for trainees, four out of eight (50.0%) stated that they incorporate this only after completion of surgical training. Nine out of 11 (81.8%) believe that robotic training should be specifically provided for trainees.

Robotic industry representatives agreed with experts and trainees that GI trainees should start bedside assisting in the first 4 years of surgical training (*n* = 11; 57.9%). A large proportion of representatives also believed that trainees should start operating on the robotic console within three to 4 years of surgical training (*n* = 8; 42.1%) and that over 30 performed cases were needed to reach the learning curve for a procedure (*n* = 6; 31.6%), which was mirrored by experts. They also believe assessment tools, particularly in the form of case logs (*n* = 15; 78.9%), subjective assessment (*n* = 14; 73.7%), and summative tools (*n* = 14; 73.7%%), were important to assess competency.

## Discussion

To our knowledge, this is the largest robotic training survey in the literature, capturing responses from European GI experts and trainees, both with and without robotic access, as well as responses from global robotic industry representatives. This pan-European survey highlighted there is a lack of implementation of structured curricula for GI trainees to achieve accreditation to become certified robotic surgeons. Desired methods for the delivery of robotic surgical training included a combination of didactic learning, simulation, virtual reality, wet lab experience (e.g. live animal or cadaveric models) and bedside assisting training, and appropriate proctoring. Currently, wet lab training is a key component of the Intuitive Technology Training Series (TR) and Training Passport Program for attending/consultant surgeons [27]. However, it was evident from our survey that access to wet lab training was particularly limited for trainees. This may be due to several reasons, including access, cost, procurement, storage, as well as religious and ethical concerns. Where possible, high fidelity 3D printed models or Kindheart simulator models should be integrated into robotic surgical training [28, 29]. The use of a dual console in robotic surgical systems was also deemed essential by both experts and trainees. It can help to simplify the proctoring system and reduce the learning curve by allowing two surgeons to operate together [30], and the pilot/co-pilot set-up also facilitates smooth instrument transfer, telestration, and supervision [31].

Experts, trainees, and industry representatives agreed that bedside assisting and console operating should occur earlier, ideally within the first 4 years of surgical training. Bedside assisting for 11–20 cases was deemed to be adequate to start operating on the console, and that performing at least 30 cases was potentially sufficient to reach or surpass the learning curve for a specific procedure. Participants also believed that surgical societies and training bodies having a greater oversight could potentially benefit their progression in robotic training. It was also evident that there is a greater need for tools or tracking assessment performance of trainees, in particular procedure-specific tools for summative and formative assessment, e.g. global evaluative assessment of robotic skills (GEARS) and objective structured assessments of technical skills (OSATS), as well as subjective assessments and case logs [32–34]. This is consistent with the findings of a recent review on this subject, which also highlighted the promising role of automated assessment tools and artificial intelligence (AI) for the objective assessment for robotic surgery, albeit they are still at the research phase [35]. There is also the emerging option of virtual training and assessment. A recent pilot study showed that virtual training of minimally invasive surgical skills using a web-based streaming platform is a potentially feasible and effective teaching and assessment method [36].

Introducing robotic surgery training earlier in the pathway may help mitigate the learning curve for trainees when they enter more advanced training. A survey conducted in 2020 at a high-volume surgical department in the USA found that 84% of attending surgeons and 80% of surgical fellows believed the optimal time for a trainee to learn robotic surgery was during general surgery residency [37]. This was also highlighted by a survey distributed to 26 general surgery residents and 18 subspecialty residents in the USA, where the majority felt that a robotic surgery curriculum should be offered during residency [38]. The Society of American Gastrointestinal and Endoscopic Surgeons (SAGES) published a consensus document in 2008 outlining desired surgical training and credentialing [39]. They stated that a structured training programme is required for those wishing to perform robotic surgery. Robotic privileges can be granted to those who have successfully completed a residency and/or fellowship programme incorporating a structured curriculum in robotic systems. On behalf of the ALSGBI, a survey was conducted investigating the current provision of robotic surgery across trusts in the UK [40]. Twenty-five out of 52 (48%) trusts stated that they had a prescribed robotic training curriculum for trainees. With forecasts of expansion, the trajectory reflected in their data suggested that 80% of trusts may have a robotic system in use by 2027.

Our survey demonstrated that a significant proportion of trainees without access would like to learn how to use the robot within the first 4 years of surgical training. One justification for this request is the likelihood of having access in their following rotation, given the rapid uptake of robotic surgery, and it is appropriate that they are prepared with the basic skills on how to use the machine. In order to accommodate this, this survey indicated that there needs to be more accredited trainers internationally, the development of a formal accreditation pathway, additional dedicated robotic training lists for trainees, and available mentorship programmes. There should be more access to robotic courses and fellowships for trainees and these should also be funded through a combination of local, regional and national programmes through a variety of funding resources if possible. Furthermore, as more hospitals buy or rent more robotic platforms, the individual operative cost is likely to significantly reduce [31]. It was also apparent that there is a greater need for industry to specifically incorporate robotic training for trainees into their own programme and this needs to be done at an earlier stage. This is important if we are to achieve standardised practices which can guide robotic training, assessment, and certification.

The Fundamentals of Robotic Surgery (FRS) was designed in 2014 to develop a curriculum of basic skills applicable to all surgical specialties for training and assessing surgeons desiring to engage in robotic surgery [41]. The curriculum, available online, contains four modules that include didactic learning, psychomotor skills, and team communication training [42]. The FRS offers a single standard of minimal skills training required to perform safe robotic surgery. However, increased participation and further validation of this curriculum, along with other emerging robotics curricula are still required [35, 43, 44]. Furthermore, the assessment of trainee competency and provision of certification of resident robotic expertise (e.g. robotic training driving licence) is an important aspect of robotic training that still needs to be addressed [13]. More recently, a centre in Germany has proposed a structured robotic surgery training programme for surgical residents in visceral surgery: the Robotic Surgery Training Curriculum (RoSTraC). This three-step curriculum consists of: (1) theoretical basics and simulation training; (2) laboratory training on the institutional robotic system and (3) structured on-patient training in the operating room [45]. As part of a prospective multicentre study, this multimodal learning approach allowed surgical residents to acquire fundamental and advanced robotic skills, significantly improving the performance of advanced four-hand robotic procedures. However, training is a dynamic process and curricula will need to be reviewed and updated as necessary. An evaluation of the upcoming robotic platforms and their use across different pathologies will ultimately lead to amendments in training. The complexity and numerous emerging robotic platforms will also make the development of training curriculum challenging, and a basic robotic skills element and a more dedicated one per platform specifications is perhaps needed.

## Future work

This survey captured diverse perspectives of experts, trainees, and industry across Europe, and gain valuable insights into training requirements, barriers, and potential solutions. Understanding the perspectives of industry representatives has also provided insight into their current policies and assessment pathways with their robotic systems. The responses to this survey will inform the subsequent ERSC Delphi process, which will involve robotic surgeons, trainees, and the extended robotic theatre team [23]. During this process, industry and patient representatives will act as external advisors to the panel to discuss if we can combine several robotic consoles into a single training curriculum. The opinions of all members of the Delphi panel will inform the development of the next phase of the project that is aiming to achieve consensus on a standardised robotic training curriculum for GI trainees that can be integrated within the training programmes. Following the development and dissemination of the ERSC robotic training curriculum, we will evaluate the uptake, impact, and efficacy of the curriculum through a comprehensive survey and other metrics.

## Limitations

This pan-European survey has some limitations that should be addressed. The survey was only a snapshot in each of these countries, with not all participants completing every single section or question of the survey. With certain questions, participants could only select a range of values, rather than provide a specific numerical value. There was also a large proportion of responses received from public teaching hospitals across Western and Southern Europe, in particular, from Germany, UK, and Italy. Although we captured responses from a diverse range of groups and specialties across Europe including experts and trainees with and without robotic access, and industry representatives, we were unable to obtain a diverse range of perspectives in terms of gender (71.7% male participants) and ethnicity (78.8% participants of white ethnic background) during our data collection process.

Furthermore, participation was voluntary, and as a result participants who have access to robotic surgery or who have strong opinions on robotic surgery may be more likely to complete the survey, leading to selection bias. This survey took a general approach to the number of procedures required for reaching or surpassing the learning curve in robotic surgery, rather than taking into account the requirements for specific procedures or procedures differing in complexity. At this stage, we also did not focus on non-technical skills or capture responses from the extended robotic theatre team (e.g. anaesthetists and theatre staff). However, this will be addressed during the Delphi process, where the ERSC Steering Committee will endeavour to ensure a broad spread of representation across key stakeholder groups, by collecting information on experience, demographic and geographical representation from the panellists. Essential non-technical skills will also be identified and incorporated into the consensus recommendations as part of this process.

## Conclusion

This pan-European survey captured views on robotic training from GI experts, trainees with and without robotic access, and industry representatives. The survey results reveal a strong opinion among stakeholders for the early integration of robotic surgery in surgical training. However, the absence of a standardised pan-European curriculum for trainees is evident, with expert training primarily driven and regulated by industry. Limited access to training centres, a shortage of trainers, and insufficient mentoring were identified as significant obstacles, with several industry representatives not specifically incorporating training for trainees in their own programmes at present. The ERSC are in the process of developing a European consensus on a robotic curriculum for GI trainees, through a Delphi process, which will integrate basic robotic skills, key clinical performance indicators, assessment tools and non-technical skills.

## Supplementary Information

Below is the link to the electronic supplementary material.

Supplementary file1 (DOCX 262 KB)
