# Supplementary material for: A pan-European survey of robotic training for gastrointestinal surgery: European Robotic Surgery Consensus (ERSC) initiative
Source: Surg Endosc. 2024 Dec 4;39(2):907–21. doi: 10.1007/s00464-024-11373-x (PMC11794360; doi:10.1007/s00464-024-11373-x)
Supplement: Supplementary file 1 — Supplementary file1 (DOCX 262 KB) [file 464_2024_11373_MOESM1_ESM.docx]

**Supplementary material**

| **Development of a European consensus in robotics for gastrointestinal surgery trainees survey** | |
| --- | --- |
| **DEMOGRAPHICS** | **Participant responses received** |
| 1. **What is your age group?** 2. 21-30 3. 31-40 4. 41-50 5. 51-60 6. 61-70 7. 71-80 8. 81-90 | 1,045 |
| 1. **What is your preferred gender identity?** 2. Female 3. Male 4. Prefer not to say 5. Other – please specify | 1,045 |
| 1. **What is your ethnic origin?** 2. Arab 3. Asian – Bangladeshi 4. Asian – Indian 5. Asian – Pakistani 6. Asian – Chinese 7. Black – African 8. Black – Caribbean 9. Mixed – Asian and White 10. Mixed – Black African and White 11. Mixed – Black Caribbean and White 12. White 13. Prefer not to say 14. Other – please specify | 1,045 |
| 1. **What is your current surgical training scheme?** 2. Full time 3. Less than full time 4. Out-of-programme research e.g. MD/PhD 5. Staff grade/SAS 6. Consultant/independent surgeon 7. Industry provider 8. Other – please specify | 1,045 |
| 1. **What is your surgical sub-specialty?** 2. General surgery 3. Upper GI 4. Hepatobiliary 5. Colorectal 6. Surgical oncology 7. Bariatric 8. Other – please specify | 1,045 |
| 1. **What stage of your surgical training/career are you?**   *All years stated in question responses relate to surgical training years (including core surgical training where applicable)*   1. Year 1-2 2. Year 3-4 3. Year 5-6 4. Year 7+ 5. Consultant/independent surgeon 6. Other – please specify. | 1,045 |
| 1. **Which country do you practice in?** | 1,045 |
| 1. **Are you a member of any surgical societies?** 2. Yes 3. No   **b. If yes, which surgical societies are you a member of?** | 1,045 |
| 1. **Is your hospital?** 2. Public (teaching) hospital 3. Public (non-teaching) hospital 4. Private (teaching) hospital 5. Private (non-teaching) hospital 6. Other – please specify | 1,045 |
| 1. **Does the hospital you practice in have a robot?** 2. Yes 3. No | 1,045 |
| 1. **At what stage are you in your robotic training?** 2. Expert/independent surgeon 3. Trainee with robotic access 4. Trainee without robotic access 5. Industry provider | 1,045 |

| **EXPERT / INDEPENDENT SURGEON** | **Participant responses received (/284)** |
| --- | --- |
| 1. **How many robotic cases have you performed?**   0/1-10/11-20/21-30/31-50/More than 50/More than 100 | 284 |
| 1. **In your current country of training, is there a dedicated training curriculum/pathway for robotic surgery for accreditation to become a consultant/independent surgeon?** 2. Yes 3. No   **b.** **If No, do you think a dedicated training curriculum/pathway for robotic surgery should become mandatory if the infrastructure is in place?** | 284 |
| 1. **If you have a training pathway, which of the following do you use?** *(select all that apply)* 2. Da Vinci Technology Training Pathway 3. Fundamentals of Robotic Surgery (FRS) 4. Fundamental Skills of Robotic-Assisted Surgery (FSRS) Training Programme 5. None 6. Other – please specify | 276 |
| 1. **Which robot do have in your institution?** *(select all that apply)* 2. DaVinci by Intuitive 3. Versius by CMR 4. Hugo by Medtronic 5. Dexter by Distalmotion 6. Senhance by Asensus 7. Not sure 8. None 9. Other – please specify. | 275 |
| 1. **Do you think a dual robotic console is essential for training?**   1 – Strongly disagree; 2 – Somewhat disagree; 3 – Neither agree nor disagree; 4 – Somewhat agree; 5 – Strongly agree | 284 |
| 1. **When do you think trainees should start virtual reality robotic training?** 2. Year 1-2 3. Year 3-4 4. Year 5-6 5. Year 7+ 6. After completion of surgical training 7. Not essential 8. Other – please specify | 284 |
| 1. **Do you think bedside training is an essential component for trainees before operating on the console?** 2. Yes 3. No   **b. If yes, for how many bedside assisting cases should surgeons have before operating on the console?**  1-10/11-20/21-30/31-50/More than 50/More than 100 | 284 |
| 1. **At what level do trainees start robotic bedside assisting in your institution?** 2. Year 1-2 3. Year 3-4 4. Year 5-6 5. Year 7+ 6. After completion of surgical training 7. Not applicable 8. Other – please specify | 284 |
| 1. **At what level should trainees start robotic bedside assisting at?** 2. Year 1-2 3. Year 3-4 4. Year 5-6 5. Year 7+ 6. After completion of surgical training 7. Other – please specify. | 284 |
| 1. **At what level do trainees start operating on the robotic console in your institution?** 2. Year 1-2 3. Year 3-4 4. Year 5-6 5. Year 7+ 6. After completion of surgical training 7. Not applicable 8. Other – please specify. | 284 |
| 1. **At what level should trainees start operating on the robotic console at?** 2. Year 1-2 3. Year 3-4 4. Year 5-6 5. Year 7+ 6. After completion of surgical training 7. Not applicable 8. Other – please specify | 284 |
| 1. **Who oversees your robotic training?** 2. The institution 3. Regional training bodies 4. National training bodies or societies e.g. RCS 5. Surgical societies e.g. EAES 6. Industry 7. Not sure 8. Other – please specify. | 284 |
| 1. **Who do you think should be overseeing robotic training for trainees?** 2. The institution 3. Regional training bodies 4. National training bodies or societies e.g. RCS 5. Surgical societies e.g. EAES 6. Industry 7. Other – please specify | 284 |
| 1. **How many cases do you think a trainee needs to perform to reach robotic surgery competency?**   1-10/11-20/21-30/31-50/51-80/81-100/More than 100 | 284 |
| 1. **Do you use tools to track or assess the performance of trainees?** *(select all that apply)* 2. Case logs 3. Subjective assessment by the trainer 4. Summative tools e.g. global evaluative assessment of robotic skills (GEARS) 5. Video assessment 6. No assessment 7. Other – please specify | 284 |
| 1. **How should competency be assessed?** *(select all that apply)* 2. Case logs 3. Subjective assessment by the trainer 4. Summative tools e.g. global evaluative assessment of robotic skills (GEARS) 5. Video assessment 6. No assessment 7. Other – please specify | 284 |
| 1. **In your opinion, how should surgical societies support robotic training?** *(please rank on a scale from 1 to 6; 6 being the most important and 1 being the least important)* 2. Mentorship programme 3. Additional robotic courses 4. Additional robotic fellowships 5. Develop simulation scenarios/team training exercises. 6. Development of performance assessment tools 7. Development of an accreditation pathway | 284 |
| 1. **Please mention up to three ways that you think robotic training can be improved.** | 181 |
| 1. **Is there any additional relevant information you would like to mention?** | 88 |
| **TRAINEES WITH ROBOTIC ACCESS** | **Participant responses received (/258)** |
| 1. **In your current country of training, is there a dedicated training curriculum for robotic surgery for accreditation to become a consultant/independent surgeon?** 2. Yes 3. No   **b. If No, do you think a dedicated training curriculum for robotic surgery should become mandatory if the infrastructure is in place?**   1. Yes 2. No | 258 |
| 1. **Are you familiar with any of the following training curriculums?** *(select all that apply)* 2. Da Vinci Technology Training Pathway 3. Fundamentals of Robotic Surgery (FRS) 4. Fundamental Skills of Robotic-Assisted Surgery (FSRS) Training Programme 5. None 6. Other – please specify | 258 |
| 1. **Which robot do have in your institution?** *(select all that apply)* 2. DaVinci by Intuitive 3. Versius by CMR 4. Hugo by Medtronic 5. Dexter by Distalmotion 6. Senhance by Asensus 7. Not sure 8. None 9. Other – please specify. | 258 |
| 1. **Do you think having a dual robotic console is essential for training?**   1 – Strongly disagree; 2 – Somewhat disagree; 3 – Neither agree nor disagree; 4 – Somewhat agree; 5 – Strongly agree | 258 |
| 1. **Do you have access to a robotic simulator on site?**     1. Yes – during working hours    2. Yes – 24 hours access    3. No    4. Other – please specify   **b. If** **No, how far would you need to travel to the nearest robotic simulator?**   1. 0 – 30 minutes 2. 31 – 60 minutes 3. 61 – 120 minutes 4. More than 120 minutes | 258 |
| 1. **Do you find online e-learning robotic training modules useful?**   1 – Strongly disagree; 2 – Somewhat disagree; 3 – Neither agree nor disagree; 4 – Somewhat agree; 5 – Strongly agree | 196 |
| 1. **Have you attended a robotic training course?** 2. Yes 3. No | 196 |
| 1. **Do you have access to robotic training in your institution?** *(select all that apply)* 2. Dedicated training lists 3. Ad hoc training lists 4. Case observation 5. Dry lab – time on robotic simulator 6. Wet lab – animal or cadaveric simulation 7. Virtual reality 8. Hub training 9. Didactic learning e.g. e-learning online courses or videos 10. None | 195 |
| 1. **Do senior trainees have access to a dedicated robotic training fellowship in your institution?**      1. Yes 2. No 3. Not sure | 195 |
| 1. **How frequently do you receive robotic training in your hospital?** 2. More than one theatre session per week 3. One theatre session per week 4. One theatre session per fortnight 5. One theatre session per month or less 6. Never | 195 |
| 1. **When do you think trainees should start virtual reality robotic training?** 2. Year 1-2 3. Year 3-4 4. Year 5-6 5. Year 7+ 6. After completion of surgical training 7. Not essential 8. Other – please specify | 195 |
| 1. **How many robotic cases have you bedside assisted in?**   0/1-10/11-20/21-30/31-50/More than 50 | 195 |
| 1. **At what level do trainees start robotic bedside assisting in your institution?** 2. Year 1-2 3. Year 3-4 4. Year 5-6 5. Year 7+ 6. After completion of surgical training 7. Other – please specify | 195 |
| 1. **At what level should trainees start robotic bedside assisting at?** 2. Year 1-2 3. Year 3-4 4. Year 5-6 5. Year 7+ 6. After completion of surgical training 7. Other – please specify | 195 |
| 1. **How many robotic cases have you performed (completed the key steps of the procedure)?**   0/1-10/11-20/21-30/31-50/More than 50 | 195 |
| 1. **At what level do trainees start operating on the robotic console in your institution?** 2. Year 1-2 3. Year 3-4 4. Year 5-6 5. Year 7+ 6. After completion of surgical training 7. Other – please specify. | 196 |
| 1. **At what level should trainees start operating on the robotic console at?** 2. Year 1-2 3. Year 3-4 4. Year 5-6 5. Year 7+ 6. After completion of surgical training 7. Other – please specify. | 195 |
| 1. **Have you faced any challenges in gaining robotic training?** *(select all that apply)* 2. Lack of robotic training lists 3. Lack of robotic accredited trainers in your hospital 4. Competition for training opportunities 5. Insufficient hands-on exposure during robotic training 6. Cost of simulator or courses 7. Great distance to travel to courses or institution with a robot 8. Failure to progress from assisting to operating 9. None 10. Other – please specify | 195 |
| 1. **Who oversees your robotic training?** 2. Your institution 3. Regional training bodies 4. National training bodies e.g. RCS 5. Surgical societies e.g. EAES 6. Industry 7. Not sure 8. Other – please specify | 195 |
| 1. **Who do you think should be overseeing your robotic training?** 2. The institution 3. Regional training bodies 4. National training bodies 5. Surgical societies e.g. EAES 6. Industry 7. Other – please specify | 195 |
| 1. **Do you use tools to track or assess the performance of trainees?** *(select all that apply)* 2. Case logs 3. Subjective assessment by the trainer 4. Summative tools e.g. global evaluative assessment of robotic skills (GEARS) 5. Video assessment 6. No assessment 7. Other – please specify | 195 |
| 1. **How should competency be assessed?** *(select all that apply)* 2. Case logs 3. Subjective assessment by the trainer 4. Summative tools e.g. global evaluative assessment of robotic skills (GEARS) 5. Video assessment 6. No assessment 7. Other – please specify | 195 |
| 1. **In your opinion, how should surgical societies support robotic training?** *(please rank on a scale from 1 to 6; 6 being the most important and 1 being the least important)* 2. Mentorship programme 3. Additional robotic courses 4. Additional robotic fellowships 5. Develop simulation scenarios/team training exercises. 6. Development of performance assessment tools 7. Development of an accreditation pathway | 195 |
| 1. **Please mention up to three ways that you think robotic training can be improved.** | 122 |
| 1. **Is there any additional relevant information you would like to mention?** | 76 |

| **TRAINEES WITH NO ROBOTIC ACCESS** | **Participant responses received (/480)** |
| --- | --- |
| 1. **Would you like to learn how to use the robot?** 2. Yes 3. No | 480 |
| 1. **When would you like to learn how to start using the robot?** 2. Year 1-2 3. Year 3-4 4. Year 5-6 5. Year 7+ 6. After completion of surgical training 7. Other – please specify. | 480 |
| 1. **How far would you be willing to travel to the nearest robotic simulator?** 2. 0 – 30 minutes 3. 31 – 60 minutes 4. 61 – 120 minutes 5. More than 120 minutes | 480 |
| 1. **What are your barriers to gaining robotic training?** *(select all that apply)* 2. Lack of robotic training lists 3. Lack of robotic accredited trainers in your hospital 4. Competition for training opportunities 5. Insufficient hands-on exposure during robotic training 6. Cost of simulator or courses 7. Great distance to travel to courses or institution with a robot 8. Failure to progress from assisting to operating 9. None 10. Other – please specify | 480 |
| 1. **In your current country of training, is there a dedicated training curriculum/pathway for robotic surgery for accreditation to become a consultant/independent surgeon?** 2. Yes 3. No   **If No, do you think a dedicated training curriculum/pathway for robotic surgery should become mandatory if the infrastructure is in place?**   1. Yes 2. No | 480 |
| 1. **Who do you think should be overseeing your robotic training?** 2. The institution 3. Regional training bodies 4. National training bodies e.g. RCS 5. Surgical societies e.g. EAES 6. Industry 7. Other – please specify | 480 |
| 1. **How should robotic surgery competency be assessed?** *(select all that apply)* 2. Case logs 3. Subjective assessment by the trainer 4. Summative tools e.g. global evaluative assessment of robotic skills (GEARS) 5. Video assessment 6. No assessment 7. Other – please specify | 479 |
| 1. **In your opinion, how should surgical societies support robotic training?** *(please rank on a scale from 1 to 6; 6 being the most important and 1 being the least important)* 2. Mentorship programme 3. Additional robotic courses 4. Additional robotic fellowships 5. Develop simulation scenarios/team training exercises. 6. Development of performance assessment tools 7. Development of an accreditation pathway | 479 |
| 1. **Please mention up to three ways that you think robotic training can be improved.** | 286 |
| 1. **Is there any additional relevant information you would like to mention?** | 159 |

| **INDUSTRY PROVIDER** | **Participant responses received (/23)** |
| --- | --- |
| 1. **Which robotic system representative are you?** 2. DaVinci by Intuitive 3. Versius by CMR Surgery 4. Hugo by Medtronic 5. Dexter by Distalmotion 6. Senhance by Asensus 7. Other – please specify | 23 |
| 1. **Do you specifically incorporate robotic training for trainees/residents in your training programme?** 2. Yes 3. No   **b. If Yes, at what stage of surgical training do you incorporate robotic training for trainees/residents in your training programme?**   1. Year 1-2 2. Year 3-4 3. Year 5-6 4. Year 7+ 5. After completion of surgical training 6. Other – please specify.   **c. If No, do you think robotic training should be provided for trainees/residents?**   1. Yes 2. No | 23 |
| 1. **When do you think trainees should start robotic bedside assisting?** 2. Year 1-2 3. Year 3-4 4. Year 5-6 5. Year 7+ 6. After completion of surgical training 7. Other – please specify. | 19 |
| 1. **When do you think trainees should start operating on the robotic console?** 2. Year 1-2 3. Year 3-4 4. Year 5-6 5. Year 7+ 6. After completion of surgical training 7. Other – please specify. | 19 |
| 1. **How many cases do you think a trainee needs to perform to reach robotic surgery competency?**   1-10/11-20/21-30/31-50/51-80/81-100/More than 100 | 19 |
| 1. **How would you recommend trainees should be assessed for robotic surgery competency?** *(select all that apply)* 2. Case logs 3. Subjective assessment by the trainer 4. Summative tools e.g. global evaluative assessment of robotic skills (GEARS) 5. Video assessment 6. No assessment 7. Other – please specify | 19 |
| 1. **Please mention up to three ways that you think robotic training can be improved.** | 12 |

**Supplementary S1.** Gastrointestinal surgery robotic training pan-European survey for experts, trainees with robotic access, trainees without robotic access and industry providers, including total number of participant responses.

|  |
| --- |
| 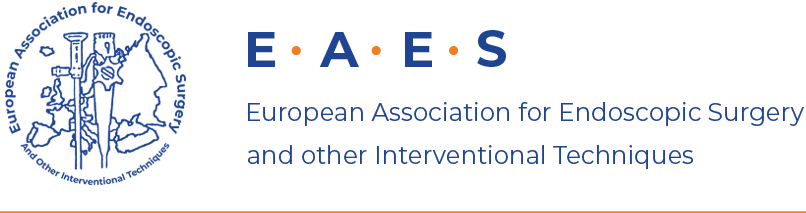 |
| **EAES Research Sandpit Survey** |
| Dear EAES family,  **This is an official survey of EAES that is endorsed by the Executive Board of the EAES.** |
| There is currently no international consensus or curriculum in robotic training for gastrointestinal (GI) surgical trainees with several issues around access, volume and a lack of experienced trainers. We are inviting you to complete the following survey, which is part of a Delphi consensus project funded by EAES, aiming to improve robotic training for GI trainees.  The survey is addressed to European GI consultant/expert surgeons, trainees/residents and global industry providers.  Please click on the link below to complete this short survey - it will only take a few minutes and it is completely anonymous. |
| \| [**Survey**](https://eaes.us14.list-manage.com/track/click?u=ad2826d541d43414cd29df0a2&id=af2ddad7bc&e=9099c661dc) \| \| --- \| \| **Survey** \| |
| Thank you very much for taking the time to complete this survey.  Kinds regards,  EAES Research Sandpit Group |

**Supplementary S2.** Example of a survey announcement distributed by the European Association for Endoscopic Surgery (EAES).

| **Section and Topic** | **Item #** | **Checklist item** | **Reported on page #** |
| --- | --- | --- | --- |
| **DESIGN** | | |  |
| Describe survey design | 1 | Describe target population, sample frame. Is the sample a convenience sample? | 6 |
| **IRB (INSTITUTIONAL REVIEW BOARD) APPROVAL AND INFORMED CONSENT PROCESS** | | |  |
| IRB approval | 2 | Mention whether the study has been approved by an IRB. | 6 |
| Informed consent | 3 | Describe the informed consent process. Where were the participants told the length of time of the survey, which data were stored and where and for how long, who the investigator was, and the purpose of the study? | 6 |
| Data protection | 4 | If any personal information was collected or stored, describe what mechanisms were used to protect unauthorised access. | 6 |
| **RECRUITMENT PROCESS AND DESCRIPTION OF THE SAMPLE HAVING ACCESS TO THE QUESTIONNAIRE** | | | |
| Open survey versus closed survey | 5 | An “open survey” is a survey open for each visitor of a site, while a closed survey is only open to a sample which the investigator knows (password-protected survey). | 6 |
| Contact mode | 6 | Indicate whether or not the initial contact with the potential participants was made on the Internet (investigators may also send out questionnaires by mail and allow for web-based data entry). | 6 |
| Advertising the survey | 7 | How/where was the survey announced or advertised? Some examples are offline media (newspapers), or online (mailing lists – If yes, which ones?) or banner advertising the survey ads (Where were these banner ads posted and what did they look like?). It is important to know the wording of the announcement as it will heavily influence who chooses to participate. Ideally the survey announcement should be published as an appendix. | 7 and S2 |
| **SURVEY ADMINISTRATION** | | | |
| Web/E-mail | 8 | State the type of e-survey (e.g. one posted on a website, or one sent out through e-mail). If it is an e-mail survey, were the responses entered manually into a database, or was there an automatic method for capturing responses? | 7 |
| Context | 9 | Describe the website (for mailing list/newsgroup) in which the survey was posted. What is the website about, who is visiting it, what are visitors normally Context looking for? Discuss to what degree the content of the website could pre-select the sample or influence the results. | N/A |
| Mandatory/voluntary | 10 | Was it a mandatory survey to be filled in by every visitor who wanted to enter the website, or was it a voluntary survey? | 6 |
| Incentives | 11 | Were any incentives offered (e.g. monetary, prizes, or non-monetary incentives such as an offer to provide the survey results)? | N/A |
| Time/Date | 12 | In what timeframe were the data collected? | 6 |
| Randomisation of items or questionnaires | 13 | To prevent biases items can be randomised or alternated. | N/A |
| Adaptive questioning | 14 | Use adaptive questioning (certain items, or only conditionally displayed based on responses to other items) to reduce number and complexity of the questions. | S1 |
| Number of Items | 15 | What was the number of questionnaire items per page? The number of items is an important factor for the completion rate. | 6 |
| Number of screens (pages) | 16 | Over how many pages was the questionnaire distributed? The number of items is an important factor for the completion rate. | S1 |
| Completeness check | 17 | It is technically possible to do consistency or completeness checks before the questionnaire is submitted. Was this done, and if “yes”, how (usually JAVAScript)? An alternative is to check for completeness after the questionnaire has been submitted (and highlight mandatory items). If this has been done, it should be reported. All items should provide a non-response option such as “not applicable” or “rather not say”, and selection of one response option should be enforced. | N/A |
| Review step | 18 | State whether respondents were able to review and change their answers (e.g. through a Back button or a Review step which displays a summary of the responses and asks the respondents if they are correct). | 7 |
| **RESPONSE RATES** | | | |
| Unique site visitor | 19 | If you provide view rates or participation rates, you need to define how you determined a unique visitor. There are different techniques available, based on IP addresses or cookies or both. | N/A |
| View rate (Ratio of unique survey visitors/unique site visitors) | 20 | Requires counting unique visitors to the first page of the survey, divided by the number of unique site visitors (not page views). It is not unusual to have view rates of less than 0.1 % if the survey is voluntary. | N/A |
| Participation rate (Ratio of unique visitors who agreed to participate/unique first survey page visitors) | 21 | Count the unique number of people who filled in the first survey page (or agreed to participate, for example by checking a checkbox), divided by visitors who visit the first page of the survey (or the informed consents page, if present). This can also be called “recruitment” rate. | N/A |
| Completion rate (Ratio of users who finished the survey/users who agreed to participate) | 22 | The number of people submitting the last questionnaire page, divided by the number of people who agreed to participate (or submitted the first survey page). This is only relevant if there is a separate “informed consent” page or if the survey goes over several pages. This is a measure for attrition. Note that “completion” can involve leaving questionnaire items blank. This is not a measure for how completely questionnaires were filled in (If you need a measure for this, use the word “completeness rate”). | N/A |
| **PREVENTING MULTIPLE ENTRIES FROM THE SAME INDIVIDUAL** | | | |
| Cookies used | 23 | Indicate whether cookies were used to assign a unique user identifier to each client computer. If so, mention the page on which the cookie was set and read, and how long the cookie was valid. Were duplicate entries avoided by preventing users access to the survey twice; or were duplicate database entries having the same user ID eliminated before analysis? In the latter case, which entries were kept for analysis (e.g. the first entry or the most recent)? | N/A |
| IP check | 24 | Indicate whether the IP address of the client computer was used to identify potential duplicate entries from the same user. If so, mention the period of time for which no two entries from the same IP address were allowed (e.g. 24 hours). Were duplicate entries avoided by preventing users with the same IP address access to the survey twice; or were duplicate database entries having the same IP address within a given period of time eliminated before analysis? If the latter, which entries were kept for analysis (e.g. the first entry or the most recent)? | N/A |
| Log file analysis | 25 | Indicate whether other techniques to analyse the log file for identification of multiple entries were used. If so, please describe. | N/A |
| Registration | 26 | In “closed” (non-open) surveys, users need to login first and it is easier to prevent duplicate entries from the same user. Describe how this was done. For example, was the survey never displayed a second time once the user had filled it in, or was the username stored together with the survey results and later eliminated? If the latter, which entries were kept for analysis (e.g. the first entry or the most recent). | N/A |
| **ANALYSIS** | | | |
| Handling of incomplete questionnaires | 27 | Were only completed questionnaires analysed? Were questionnaires which terminated early (where, for example, users did not go through all questionnaire pages) also analysed? | 7 |
| Questionnaires submitted with an atypical timestamp | 28 | Some investigators may measure the time people needed to fill in a questionnaire and exclude questionnaires that were submitted too soon. Specify the timeframe that was used as a cut-off point, and describe how this point was determined. | N/A |
| Statistical correction | 29 | Indicate whether any methods such as weighting of items or propensity scores have been used to adjust for the non-representative sample; if so, please describe the methods. | 7-8 |

**Supplementary S3.** CHERRIES checklist. N/A, not applicable.

**A**

**B**

**C**

**Supplementary S4.** (A) Experts, (B) trainees with robotic access and (C) trainees without robotic access ranked as being least important [1] to most important [6] in response to how should surgical societies support robotic training.
